# The role of pore fluids in supershear earthquake ruptures

**DOI:** 10.1038/s41598-022-27159-x

**Published:** 2023-01-09

**Authors:** Pedro Pampillón, David Santillán, Juan C. Mosquera, Luis Cueto-Felgueroso

**Affiliations:** 1 Department of Civil Engineering: Hydraulics, Energy and Environment, Universidad Politécnica de Madrid, Madrid, Spain; 2 Department of Continuum Mechanics and Theory of Structures, Universidad Politécnica de Madrid, Madrid, Spain

**Keywords:** Seismology, Civil engineering

## Abstract

The intensity and damage potential of earthquakes are linked to the speed at which rupture propagates along sliding crustal faults. Most earthquakes are sub-Rayleigh, with ruptures that are slower than the surface Rayleigh waves. In supershear earthquakes, ruptures are faster than the shear waves, leading to sharp pressure concentrations and larger intensities compared with the more common sub-Rayleigh ones. Despite significant theoretical and experimental advances over the past two decades, the geological and geomechanical controls on rupture speed transitions remain poorly understood. Here we propose that pore fluids play an important role in explaining earthquake rupture speed: the pore pressure may increase sharply at the compressional front during rupture propagation, promoting shear failure ahead of the rupture front and accelerating its propagation into the supershear range. We characterize the transition from sub-Rayleigh to supershear rupture in fluid-saturated rock, and show that the proposed poroelastic weakening mechanism may be a controlling factor for intersonic earthquake ruptures.

## Introduction

Ground motion during earthquakes is due to the radiation of elastic energy from rapidly sliding faults. Rupture nucleates at a weak patch, and then propagates over long sections of the fault. Some of the most destructive earthquakes on record are believed to have reached supershear rupture speeds^1–7^. The occurrence of supershear earthquakes is compatible with the classical theory of fracture mechanics^8–12^, and their damaging potential stems from a distinctive pattern of velocities and accelerations associated with intense pressure concentration near the rupture front^13–17^. Admissible rupture regimes have been derived in analogy with the propagation of shear cracks, and are classified by comparing the rupture speed, ${\bar{V}}_{R}$, with the speed of body and surface waves: the compressional P-wave ($C_{P}$), the shear S-wave ($C_{S}$), and the surface Rayleigh wave ($C_{R}$ $\approx$ 0.92 $C_{S}$). Most natural earthquakes are sub-Rayleigh, ${\bar{V}}_{R}$ < $C_{R}$, approaching the Rayleigh speed in the limit of vanishing shear strength^18^. Steady rupture propagation in the range between the Rayleigh and shear wave speeds for mode II fractures, $C_{R}$ < ${\bar{V}}_{R}$ < $C_{S}$, is precluded because it implies a negative energy flux to the leading front^12,19^, albeit steady rupture speeds between the Rayleigh wave speed and Eshelby speed are admissible in a mix-mode fracture^20,21^. The stable intersonic regime includes rupture speeds between the Eshelby speed, $\sqrt{2}C_{S}$, and $C_{P}$^9^. The Eshelby speed arises in theoretical models either as a boundary between real-solution and unstable zones^9,22^, or as an asymptotic speed for well-developed supershear ruptures^11,23^.

The hypothesis of rupture speeds that accelerate into the supershear range has been instrumental to explain unconventional patterns of strong ground motion over the past two decades^1–7,13^, and yet our understanding of the controlling mechanisms leading to supershear rupture in crustal earthquakes remains fragmented. The analogy between earthquakes and growing shear cracks has led to the experimental confirmation that intersonic^23–26^, and even supersonic^16^ ruptures are possible. Supershear speeds are often linked to stress concentrations along faults, caused by geometric features such as kinks, corners and barriers^27–32^, or by fault heterogeneity and the presence of rough patches^33–36^. Off-fault plasticity and damage^37–39^, bimaterial interfaces^40^ and the presence of stronger fault segments^41^ have also been shown to promote sub-Rayleigh to supershear transitions. Supershear is favored by low frictional resistance in the sliding patch, which is ultimately governed by fault constitutive behaviour^10,34,42–44^. The role of the different controlling mechanisms is typically understood in the context of the Burridge-Andrews mechanism, according to which supershear cracks arise from the generation and propagation of a daughter crack ahead of the tip, due to stresses moving at intersonic speeds in this region^9,14,42^. The standard criterion to classify rupture speeds is the Andrews seismic ratio: a ratio of initial to residual strength that determines whether sub-Rayleigh ruptures will accelerate to supershear and, if so, what is the critical rupture length for the transition to occur^9,14,45^.

In this work we propose that the presence and compressibility of the pore fluids, and the coupled rock-fluid response during coseismic rupture, are essential to understand rupture speed. Fluids fill the pore space of crustal rocks, and pore pressures are essential to understand fault stability. However, poroelastic effects are often neglected in the analysis and interpretation of earthquake rupture speeds. Fast deformations during slip propagation lead to an undrained pore pressure response, which may be very intense at the compressional rupture front, enhancing frictional weakening, increasing shear stresses and promoting supershear rupture propagation. The appearance of large undrained overpressures due to fast slip events has been previously characterized for earthquake ruptures at bimaterial interfaces^46,47^, in homogeneous poroelastic media^48^, and in landslides^49^. The open question addressed in this study is under what fluid and rock properties and geomechanical conditions may the poroelastic coseismic weakening mechanism be a driver of rupture speed, potentially promoting supershear speeds.

**Figure 1:**
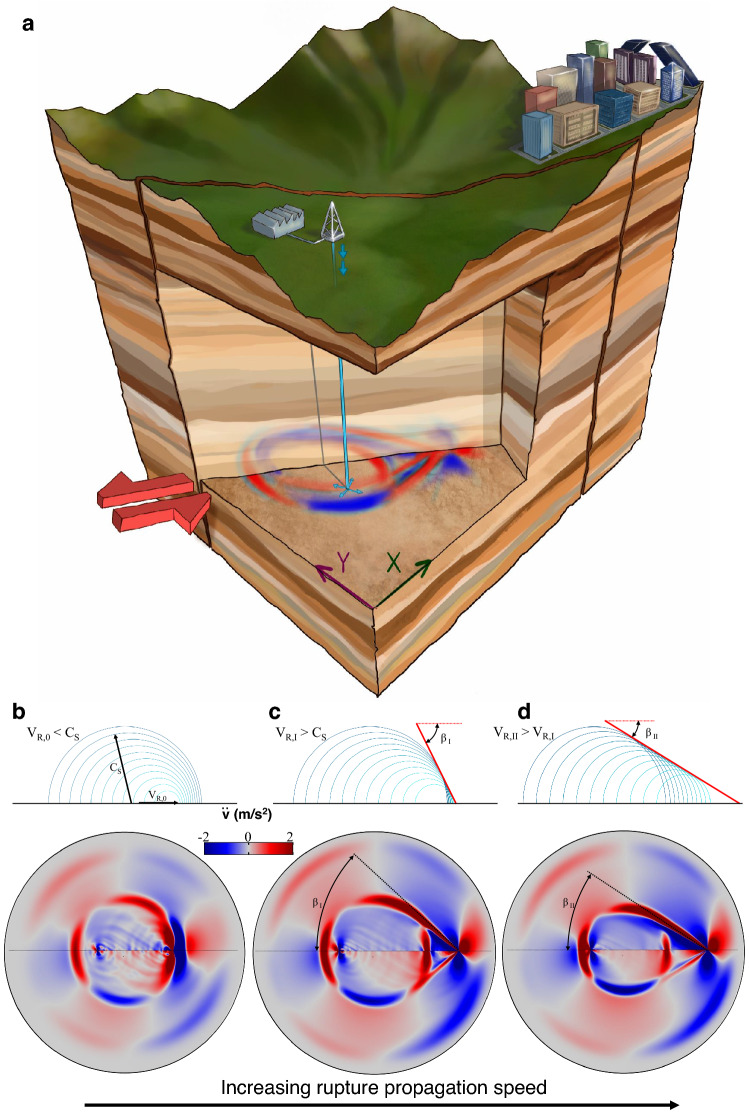
(**a**) We simulate earthquake ruptures on a velocity-weakening strike-slip fault, and study the impact of rock-fluid coupled responses on rupture speed (see Supplementary Information). (**b**–**d**) Solid acceleration patterns reveal the transition from sub-Rayleigh to supershear rupture. In supershear earthquakes, a Mach cone emerges behind the propagating rupture front (illustrated here through snapshots of the solid accelerations at a horizontal plane in **c** and **d**). Above each plot of accelerations, we show a conceptual description of the superposition of radiating waves during sub-Rayleigh (**b**) and supershear (**c** and **d**) ruptures.

We use numerical simulations of dynamic rupture in saturated poroelastic media to study the impact of rock-fluid coupled response on the transition from sub-Rayleigh to supershear rupture (Fig. 1. See Supplementary Information, Section 1). Our simulations explore the poroelastic control on rupture speed in fully saturated media, and characterize the conditions for supershear propagation in terms of system compressibility, rock mechanical properties and pre-existing stress state. The total system compressibility provides the connection among pore fluid properties, rock types and supershear earthquakes, while rock stiffness and confinement stresses constrain the range of depths for which the poroelastic mechanism may be a controlling factor for intersonic ruptures in natural earthquakes.

## Undrained poroelastic response as a coseismic weakening mechanism

The link between mechanical deformations and pore pressures in permeable rocks can be understood using Terzhagi’s principle of effective stress and Biot’s theory of poroelasticity^50,51^. During slow deformations, pore pressures accommodate changes in volumetric strain diffusively and non-locally, through fluid flow. When deformations are too fast for pressures to equilibrate via fluid drainage, strains are locally balanced by the system compressibility, so that changes in volumetric strain, $\epsilon _{\text {vol}}$, lead to pressure changes, such as: $\Delta p$ $=$ $-\frac{\alpha _B}{S_{\epsilon }}$ $\Delta$ $\epsilon _{\text {vol}}$. The storativity, or total system compressibility, $S_{\epsilon }$ $=$ $\phi \chi _f$ $+$ $\frac{(\alpha _B-\phi )(1-\alpha _B)}{K}$, includes storage via fluid compressibility, $\chi _f$, and through compressibility of the rock skeleton and solid grains, $\frac{(\alpha _B-\phi )(1-\alpha _B)}{K}$, where $\phi$ is the rock porosity and $\alpha _B$ is the Biot coefficient, $\alpha _B$ $=$ 1 − $K/K_s$. The latter compares the bulk modulus of the solid grains, $K_s$, with that of the porous medium, *K* $=$ *E*/3(1 − $2\nu )$, which depends on the Young modulus, *E*, and Poisson’s ratio, $\nu$. Coseismic rupture induces quick deformations and undrained pressure changes in the near-fault region and, in particular, at the propagating rupture fronts. The undrained pressure response can be interpreted in terms of stress change, by relating volumetric strain and mean total stress, $\Delta \epsilon _{\text {vol}}$ $=$ $\frac{1}{K}(\Delta \sigma _m$ $+$ $\alpha _B\Delta p)$, which leads to pressure changes during coseismic rupture propagation: $\Delta p$ $=$ $-B \Delta \sigma _m$, where *B* is the Skempton’s coefficient^52^, *B* $=$ $\alpha _B/(\alpha _{B}^2$ $+$ $K S_{\epsilon })$, and $\Delta \sigma _m$ $=$ $(\Delta \sigma _x$ $+$ $\Delta \sigma _y$ $+$ $\Delta \sigma _z)/3$ is the mean stress change. The impact of undrained overpressure on fast slip events has been previously characterized for earthquake ruptures in poroelastic media with homogeneous^48^ and bimaterial interfaces^46,47^, and in landslides^49^.

**Figure 2:**
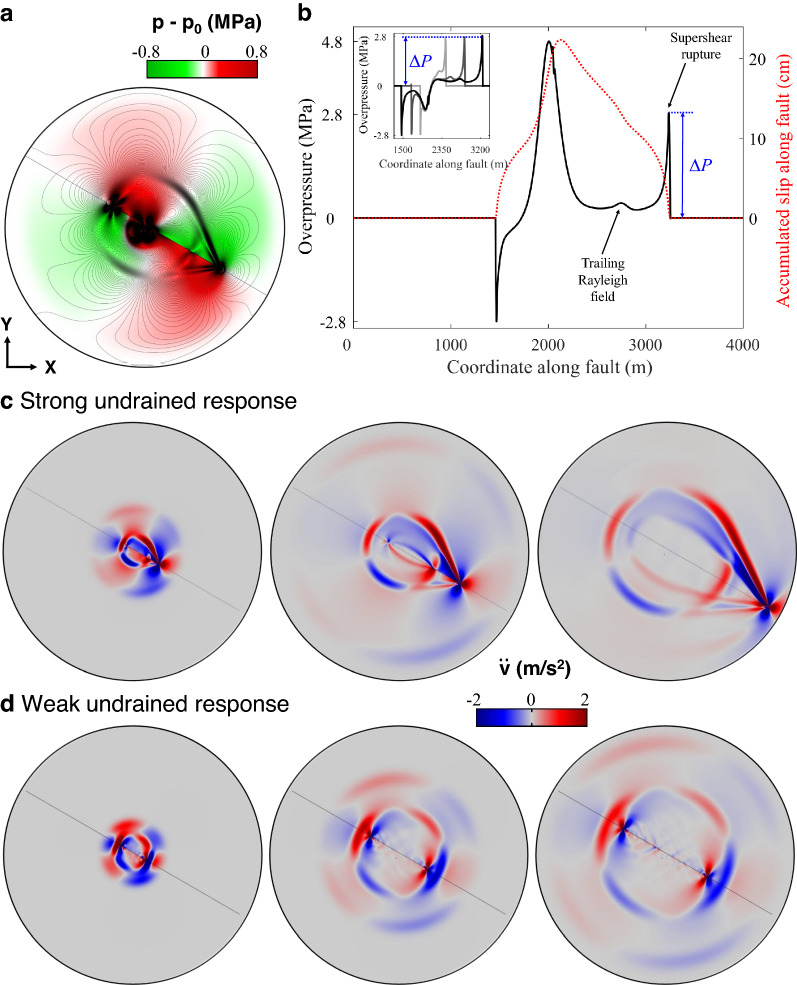
Undrained poroelastic response as a coseismic weakening mechanism. With the simulation setup of Fig. 1, we illustrate the influence of pore pressure response on rupture propagation speed. (**a**) Snapshot of coseismic pore pressure changes and contours of shear stress during rupture propagation. The reference pressure state, $p_0$, is computed right before nucleation. Fast fault slip propagation leads to a pattern of compressions and extensions that induce sudden changes in pore pressure around the fault (red, increases; green, decreases). (**b**) Undrained pressure changes are particularly strong and sharp at the rupture tip, as revealed by the profiles of pore overpressure (solid black line) and accumulated slip (dotted red line) along one side of the fault. The undrained overpressure at the detachment front, $\Delta P$, remains nearly constant over time (inset of **b**). (**c**,**d**) To highlight the impact of undrained response on earthquake rupture speed, we show the effect of changing Biot’s coefficient on the type of rupture propagation, while keeping the other parameters constant. In the case of $\alpha _B$ $=$ 0 (**d**), which neglects poroelastic coupling, rupture is symmetric and sub-Rayleigh, whereas for $\alpha _B$ $=$ 0.95 (**c**), the ensuing rupture is asymmetric, with one of the edges propagating at supershear speed.

Processes that reduce frictional strength, $\tau _s$, near the front tip promote faster ruptures, even in the sub-Rayleigh range^18^. We propose that the undrained poroelastic response during rupture propagation is a mechanism of coseismic weakening (Fig. 2). Fast slip leads to a nearly antisymmetric pattern of compressions and extensions that induce a sudden increase in pore pressure at the fault tips (Fig. 2a). Coseismic undrained overpressures weaken the fault because they reduce the effective normal stress, $\Delta \sigma _n'$ $=$ $\sigma _n$ $+$ $\Delta p$, and hence decrease the fault frictional strength, which under the cohesionless assumption is given by $\tau _s$ $=$ $-\mu \sigma _n'$. The magnitude of the sharp overpressure front at the propagating rupture tip, $\Delta P$ (Fig. 2b), depends on the system compressibility, stress released during rupture, and on the degree of poroelastic coupling, which is a property of the rock that varies with depth and confinement conditions. For the same fluid and rock system parameters, and geomechanical conditions, ruptures on poroelastic media may switch from sub-Rayleigh to supershear depending on the extent of the coseismic undrained response (Fig. 2c,d).

To elucidate the connection between the properties of pore fluids, rock matrix and geomechanical constraints on rupture speed, we study dynamic ruptures in poroelastic media with several system compressibilities, confinement scenarios and rock mechanical properties. We compare the measured rupture speeds with the shear wave speed, $C_{S,0}$ $=$ $\sqrt{G/\rho _b}$, where $\rho _b$ is the bulk density of the fluid-rock system and $G=\frac{E}{2(1+\nu )}$ is the shear modulus of the rock. The ratio between the Rayleigh and shear wave speeds for elastic media is a function of the Poisson ratio^11^, $\frac{C_{R,0}}{C_{S,0}}$ $=$ $\frac{0.862+1.14\nu }{1+\nu }$. As a reference, $C_{R,0}/C_{S,0}$ $=$ 0.9176 for $\nu$ $=$ 0.25. Wave celerities and the relationships between them vary for elastic, poroelastic, and poroviscoelastic media. We interpret their effect in the context of the Gassmann wave celerities for saturated rocks^53^ with undrained shear and bulk moduli given by $G_{\text {sat}}$ $=$ *G* and $K_{\text {sat}}$ $=$ $K+\alpha _B^2/S_{\epsilon }$, respectively. The modified compressional and shear wave speeds, $C_{P,G}$ and $C_{S,G}$, are respectively given by $C_{P,G}$ $=$ $\sqrt{(K_{\text {sat}}+\frac{4}{3}G_{\text {sat}})/\rho _b}$ and $C_{S,G}$ $=$ $C_{S,0}$.

The undrained pressure change induced by coseismic slip is antisymmetric and discontinuous across a sealing fault (Fig. 2a), which raises a fundamental and practical question about how to define an equivalent fault pressure along the fault. This equivalent fault pressure is used to evaluate frictional strength at each point along the fault (See Supplementary Information, Section 1). A rigorous definition of equivalent fault pressures at the meter and kilometer scale requires upscaling of small-scale poromechanical processes inside the fault zone. When there is a large pressure jump across the fault, as in the case of a sealing fault or after a sudden change in pore pressure due to undrained poroelastic response, micromechanical simulations have shown that the correct equivalent fault pressure is the maximum of both sides, and that using the arithmetic average actually leads to incorrect predictions of fault stability^54,55^. Equivalent pressures can also be defined as a weighted average between the pore pressures on both sides of the fault, the weights being given by a function of permeabilities and storage coefficients^46,47^. In this work, we adopt the criterion of maximum pressure of both fault sides^54,55^, so that the pressure used to compute fault frictional strength is $p = \max (p^-,p^+)$, where $p^{\pm }$ denotes pore pressure on either side of the fault when there is a pressure discontinuity on the fault at the modeling scale. The effective contact pressure at the fault, $\sigma _n'$, is given by $\sigma _n'=p-T_n$, with $T_n$ being the contact pressure between the fault edges (compressive pressures are positive). The fault remains locked when the shear stress acting on the fault is lower than the frictional strength, $\tau _f$ ; otherwise, it slips.

We neglect dilatancy during coseismic rupture, which could in principle modify the storativity of the fault zone^56–59^. We also neglect other coseismic weakening mechanisms such as flash heating^60,61^. Laboratory observations (velocity-stepping experiment under drained conditions) show that changes in slip velocity promote dilatancy^56,62^. Literature shows that for seismogenic depths, shear-induced dilatancy may be of sufficient magnitude to fully depressurize pore fluid, thereby inhibiting seismic rupture nucleation or propagation^57,63^. However, the exact conditions that lead to compaction or dilation during nucleation or rupture remain poorly understood^59,64^.

## Results: impact of fluid compressibility and rock properties on supershear ruptures

**Figure 3:**
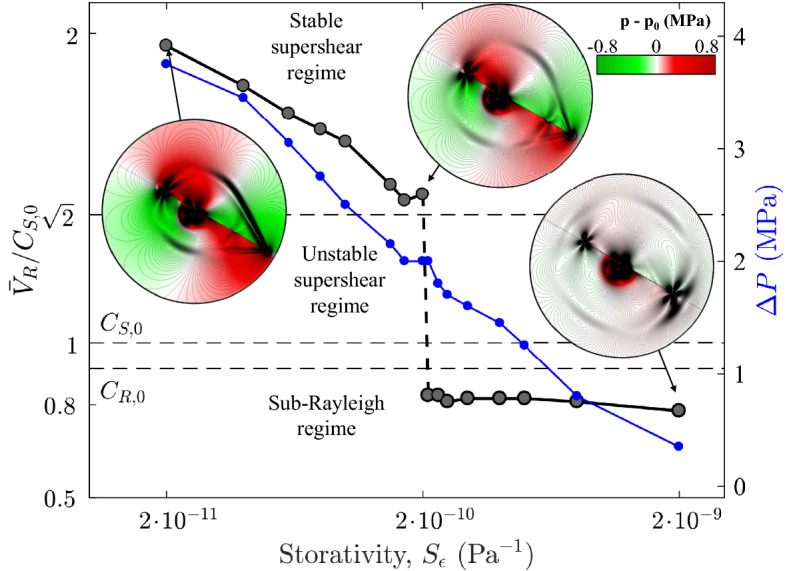
Effect of rock-fluid system compressibility on rupture speed. With the simulation setup of Fig. 1, we vary the specific storage coefficient, $S_{\epsilon }$, which controls the extent of the undrained overpressure at the detachment front, $\Delta P$. We plot the normalized rupture velocity, ${\bar{V}}_R/C_{S,0}$ (black line, left axis), and the undrained overpressure at the rupture front, $\Delta P$ (blue line, right axis), against $S_{\epsilon }$. For rather incompressible systems (small values of $S_{\epsilon }$), the overpressure is significant and the rupture is supershear. As the system storage capacity increases, thus reducing the coseismic undrained overpressure, propagation speed decreases. Beyond a threshold value of $S_{\epsilon }$, the rupture front velocity falls within the sub-Rayleigh range. This abrupt parametric shift is compatible with the existence of a range of unstable rupture speeds: if the fault is large enough, ruptures would either become asymptotically sub-Rayleigh, with ${\bar{V}}_R$ $\approx$ $0.8C_S$, or accelerate to supershear with rupture speeds larger than the Eshelby speed, $\sqrt{2}C_{S,0}$. The reported rupture speeds are calculated as the average during the rupture (see Supplementary Information, Section 3).

We first explore the impact of fluid compressibility by simulating ruptures in rocks with various storage coefficients, $S_{\epsilon }$, and plotting the normalized rupture propagation speed, ${\bar{V}}_R/C_{S,0}$, as a function of $S_{\epsilon }$ (Fig. 3). We set $\alpha _B$ $=$ 1 and $\phi$ $=$ 0.1, so that the storage coefficient is controlled by the fluid compressibility alone, $S_{\epsilon }$ $=$ $\phi \chi _f$. All other model parameters remain unchanged. When the system storage capacity is large, ruptures tend to remain in the sub-Rayleigh regime, and coseismic overpressures, $\Delta p$, are relatively small (Fig. 3). As the system compressibility decreases (smaller values of $S_{\epsilon }$), undrained overpressures during rupture are large enough so that coseismic weakening triggers supershear propagation. Parametrically, the shift from sub-Rayleigh to supershear rupture is sharp, ocurring at a specific value of $S_{\epsilon }$ that depends on confinement and rock stiffness. This abrupt shift is compatible with the existence of a range of unstable rupture speeds: if the fault is large enough, ruptures would either become asymptotically sub-Rayleigh with ${\bar{V}}_R$ $\approx$ $0.8C_S$, or accelerate to supershear with rupture speeds larger than the Eshelby speed, $\sqrt{2}C_{S,0}$.

The connection between specific storage and rupture speed points to the key role of fluid and solid compressibilities in understanding supershear earthquakes. It is worth mentioning the compressibilities of water ($\chi _f$ $\approx$ 4 $\cdot$ $10^{-10}$ Pa$^{-1}$) and supercritical CO$_2$ ($\chi _f$ $\approx$ 4 $\cdot$ $10^{-8}$ Pa$^{-1}$). For nearly incompressible rocks $\alpha _B$ $\approx$ 1, and the total compressibility is controlled by the fluid compressibility. In general the rock compressibility is not negligible, and the Biot coefficient decreases with depth^65,66^ and with increasing both confining and effective stress level^67^.

**Figure 4:**
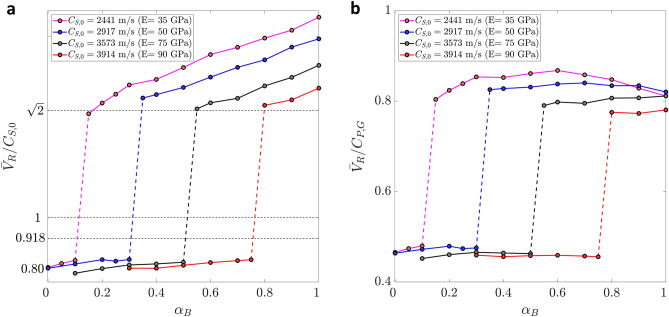
Impact of rock poromechanical properties on rupture speed. (**a**) The transition from sub-Rayleigh to supershear ruptures occurs at larger values of the Biot coefficient as the material becomes stiffer. The normalized rupture speed, $\overline{V}_{R}$/$C_{S,0}$ indicates that ruptures tend to be sub-Rayleigh when poroelastic coupling is weak (smaller $\alpha _B$), while strong coupling (larger $\alpha _B$) favours supershear ones. For given confinement stresses, rock stiffness controls the transition from sub-Rayleigh, with ${\bar{V}}_R$ $\approx$ $0.8C_S$, to supershear beyond the Eshelby speed, ${\bar{V}}_R$ $\ge$ $\sqrt{2}C_{S,0}$. (**b**) Using the same results as in (**a**), we normalize rupture speeds using the Gasmann modification of the compressional wave speed, $C_{P,G}$. Ruptures stabilize around a normalized speed of 0.8. The confinement conditions are $(\sigma _x,\sigma _y)$ $=$ (130 MPa, 50 MPa).

**Figure 5:**
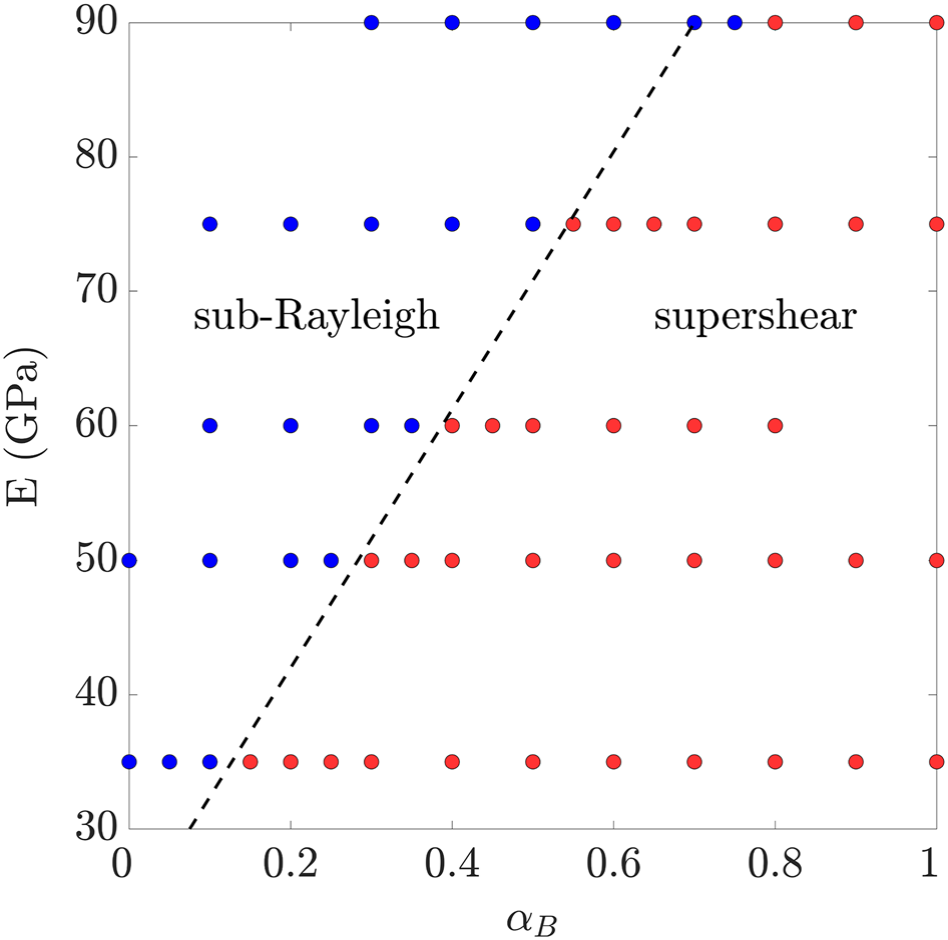
Phase diagram of sub-Rayleigh and supershear in the *E*-$\alpha _B$ space, using the data shown in Fig. 4. Red dots are supershear cases, and blue dots are sub-Rayleigh ones. We also plot a tentative phase boundary (dashed black line).

We then explore the impact of rock compressibility and stiffness by varying the Biot coefficient and the drained bulk modulus of the rock, *K*, for given confinement conditions (Fig. 4). We observe a sharp behaviour in the relative rupture propagation speed, ${\bar{V}}_{R} / C_{S,0}$, as the Biot coefficient decreases, so that weaker poroelastic couplings (smaller $\alpha _B$) lead to sub-Rayleigh ruptures, while large values promote supershear ones (Fig. 4a). The Biot coefficient is known to decrease with depth, as the compressibility of the rock matrix becomes comparable to that of the solid grains^65^. This suggests that the poroelastic coseismic weakening mechanism could be more relevant for shallow ruptures rather than deep ones, and for rocks with larger Biot coefficient. Supershear ruptures are favoured in soft media subjected to large confinement stresses (Fig. 4b): for given confinement stresses, rock stiffness controls the transition from sub-Rayleigh, with ${\bar{V}}_R$ $\approx$ $0.8C_S$, to supershear beyond the Eshelby speed, ${\bar{V}}_R$ $\ge$ $\sqrt{2}C_{S,0}$. (b) Using the same results as in panel (a), we normalize rupture speeds using the Gassmann modification of the compressional wave speed, $C_{S,G}$. Ruptures stabilize around a normalized speed of 0.8 (Fig. 4**b**). The phase diagram of sub-Rayleigh and supershear ruptures as a function of Young’s modulus and Biot coefficient reveals a linear boundary between the two rupture speed regimes (Fig. 5).

## Interpretation in the context of Andrews’ theory of critical length for supershear transition

In the classical Andrews theory of ruptures in elastic media with slip-weakening faults^9^, the rupture length for supershear transition can be approximated as^14^: *L* $=$ $9.8(1.77-\text {S})^{-3}L_c$, where the seismic ratio, S, is given by^9,14^: $\text {S}$ $=$ $\frac{\tau _p-\sigma _{xy}^{0}}{\sigma _{xy}^{0}-\tau _r}$, and the critical crack length for the supershear transition is^14^: $L_c$ $=$ $\frac{G(\tau _p-\tau _r)D_c}{\pi (1-\nu )(\sigma _{xy}^{0}-\tau _r)^2}$, where *G* is the bulk shear modulus, $D_c$ is the critical slip weakening distance—a property of the fault’s friction law—, and $\tau _p$, $\tau _r$, and $\sigma _{xy}^{0}$ are, respectively, the static and dynamic frictional strength and initial shear traction acting on the fault. The strengths $\tau _p$, $\tau _r$ are often interpreted as the initial and residual strengths after the rupture front has passed, in the context of slip weakening faults, $\tau _p$ $=$ $\mu _0 \sigma _n^{0}$ and $\tau _r$ $=$ $\mu _d \sigma _n^{0}$, where $\mu _0$ and $\mu _d$ are the static and dynamic friction coefficients, respectively, and $\sigma _n^{0}$ is the normal stress applied on the fault, which is assumed to be constant in the above expression. This ratio compares initial and residual frictional strength during rupture propagation. Andrews noted that a seismic ratio $\text {S}$ < 1.77 assures the transition from sub-Rayleigh to supershear regime^9,14^. We define an analogous seismic ratio $S'$ as $S'=S1/S2'$, with $S1=\mu _s \sigma _n'-\tau _a$ and $S2'=\tau _a-\mu _d \sigma _n'$. In this expression $\mu _s$ and $\mu _d$ are the static and dynamic friction coefficients, respectively, $\sigma _n'$ is the effective normal stress, and $\tau _a$ is the applied shear stress. We calculate $\sigma _n'$ by using the time-averaged value of the effective stress at the propagating rupture front (see Supplementary Fig. S4).

**Figure 6:**
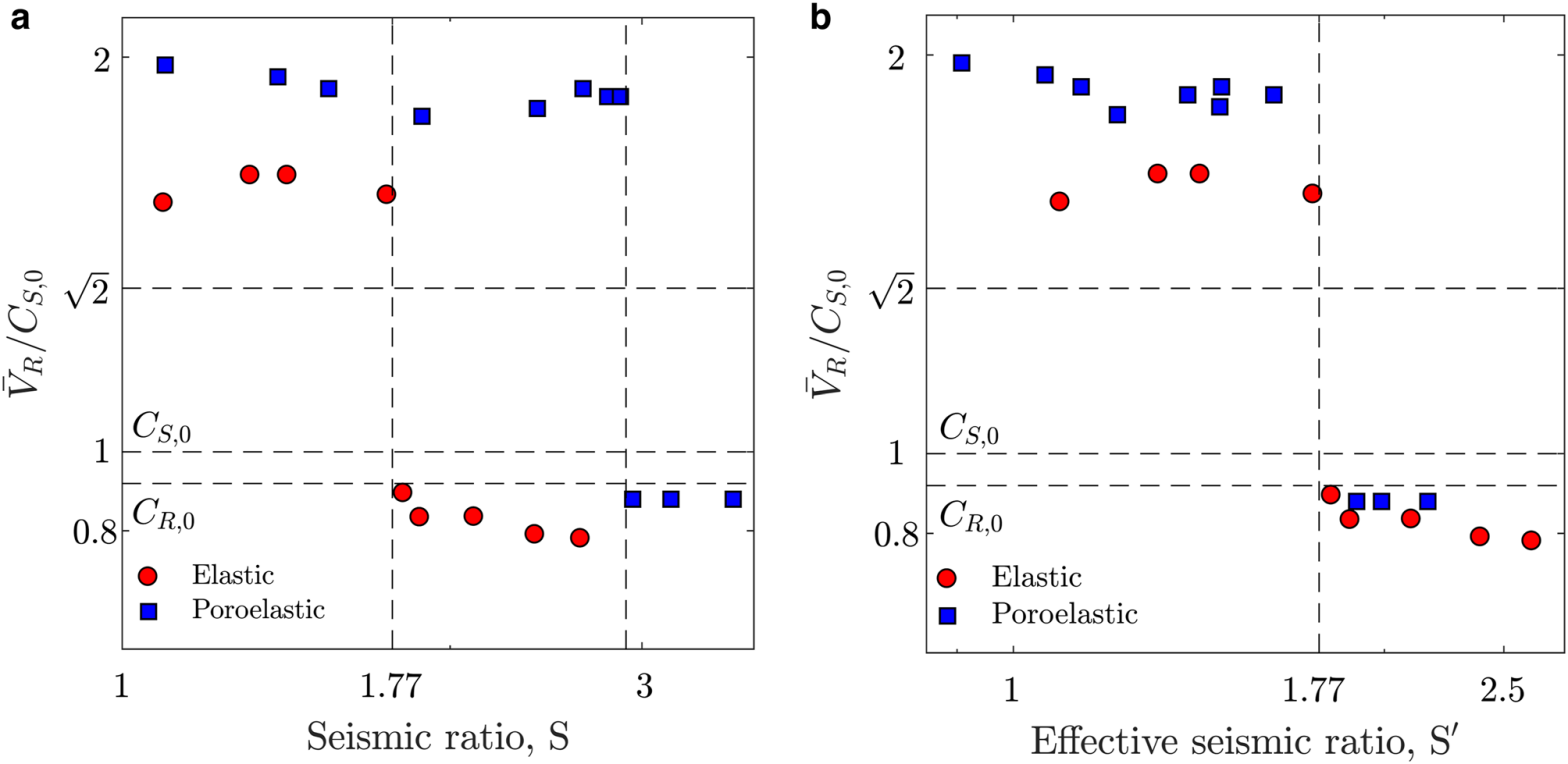
Interpretation of supershear ruptures in poroelastic media through an effective seismic ratio. We simulate ruptures in a slip-weakening, strike-slip fault model (see Supplementary Material, Section 2, for the model setup). (**a**) We perform simulations with several dynamic friction coefficients, and verify that the transition for ruptures in elastic media occurs at the Andrews seismic ratio of 1.77 (red circles), while the transition for poroelastic media ($\alpha _B$ $=$ 0.95) occurs at a significantly larger value (blue squares). (**b**) By expressing the seismic ratio using *effective*, rather than total normal stresses (see Supplementary Material, Section 4, for the calculation of the effective residual strength), we recover the transition at an effective seismic ratio $\text {S}'$ $\approx$ 1.77.

We analyze whether the Andrews theory, which serves as the basic theoretical framework to understand supershear ruptures in elastic media, is valid for poroelastic rocks (Fig. 6). We replicate the conditions leading to Andrews’ result, by simulating tectonically-driven ruptures in a slip-weakening strike-slip fault model, for which is easy to characterize the stresses in the seismic ratio^9^ (see Supplementary Material, Section 2, for the model setup). We perform simulations with different dynamic friction coefficients, and verify that the transition for ruptures in elastic media occurs at the Andrews seismic ratio of 1.77 (Fig. 6a). The transition for poroelastic media ($\alpha _B$ $\approx$ 1) occurs at a significantly larger value. By expressing the seismic ratio using effective, rather than total, normal stresses (see Supplementary Material, Section 4, for the calculation of effective residual strength), we recover the transition at an *effective* seismic ratio $\text {S}'$ $=$ 1.77. While the strict equivalence between elastic and poroelastic media based on an effective seismic ratio cannot be generalized, because of the simplicity of the slip-weakening law, it provides a straightforward expression to incorporate pore fluids into the theory of earthquake rupture speed.

## Conclusion: role of poroelasticity in supershear earthquake ruptures

One of the features of some highly destructive earthquakes is their supershear rupture propagation, with velocities faster than the shear wave speed that typically lead to large magnitude events. The intensity and the patterns of strong ground motion for supershear earthquakes have been shown to be inherently different from those of sub-Rayleigh ones^13,14^, calling for a need to elucidate the controlling factors behind rupture speed to understand risk^28^. In this Article, we highlight the role of pore fluids in the coseismic weakening mechanism that promotes the transition to supershear earthquake ruptures. The presence of nearly incompressible pore fluids and porous rocks is an essential feature to understand such ruptures. By using numerical simulations we demonstrate that, as a result of undrained deformations during the rupture propagation, poroelastic coupling leads to a pore pressure increase at the rupture front hence promoting the transition to supershear rupture. We have characterized the impact of some hydro-mechanical and frictional parameters on this coseismic weakening mechanism. In this regard, the pore fluid compressibility plays a paramount role in the transition: highly compressible fluids boost sub-Rayleigh ruptures, while nearly incompressible fluids and rocks produce a larger poroelastic response, promoting supershear ruptures. Depending on conditions, a medium saturated with water may lead to a supershear earthquake, while if saturated with supercritical CO$_{2}$ the rupture would be sub-Rayleigh. Our results indicate that the classical Andrews theory, used to understand the occurrence of supershear ruptures, needs to be revised to account for undrained poroelastic effects. A simple approach is to reinterpret the seismic ratio in terms of effective stresses. We demonstrate that the value of 1.77 proposed as the threshold between the sub-Rayleigh and the supershear transition is valid for mode II shear cracks with slip-weakening friction if the residual strength is calculated by using effective normal stresses. Our results show that there is an abrupt transition from sub- to super-shear rupture regimes as the effect of the undrained coseismic response decreases. In particular, the compressibility of resident and injected fluids around the fault emerges as a key parameter to understand the occurrence of supershear ruptures in both natural and induced earthquakes.

## Methods

Complete Methods are provided as Supplementary Information. We provide details on the numerical simulation setup used to obtained the results discussed in the main text. We present the mathematical model of dynamic earthquake rupture in poroelastic media (Section 1), followed by a description of the model setup for the two types of triggering mechanisms: fluid injection near the fault and tectonic loading (Section 2). Section 3 describes the calculation used to estimate the rupture front propagation speed from the simulation results, and Section 4 describes the calculation of the seismic ratio from the simulation results.

## Supplementary Information

Supplementary Information.

## Data Availability

The datasets generated and analysed during the current study are available from the corresponding author on reasonable request.
