# Supplementary material for: The role of pore fluids in supershear earthquake ruptures
Source: Sci Rep. 2023 Jan 9;13:398. doi: 10.1038/s41598-022-27159-x (PMC9829726; doi:10.1038/s41598-022-27159-x)
Supplement: Supplementary file 1 — Supplementary Information. [file 41598_2022_27159_MOESM1_ESM.pdf]

Supplementary Information for: The role of pore fluids on supershear earthquake ruptures, by

P. Pampillón, D. Santillán, J. C. Mosquera and L. Cueto-Felgueroso

Summary

In this Supplementary Information we provide details on the numerical simulation setup used to obtained the results discussed in the main text. We present the mathematical model of dynamic earthquake rupture in poroelastic media (Section 1), followed by a description of the model setup for the two types of triggering mechanisms: fluid injection near the fault and tectonic loading (Section 2). Section 3 describes the calculation used to estimate the rupture front propagation speed from the simulation results. Section 4 discusses the impact of grid resolution on rupture speed, and Section 5 describes the calculation of the seismic ratio from the simulation results.

S1. Mathematical model for dynamic ruptures in poroelastic media

The mass conservation equation for the fluid is given by:

$$S_\epsilon \frac{\partial p}{\partial t} + \alpha_B \frac{\partial \varepsilon_v}{\partial t} = \nabla \cdot \left(\frac{k}{\eta_f} \left(\nabla p - \rho_f \mathbf{g} \right) \right), \tag{1}$$

written in terms of the pore pressure, p, and volumetric strain, ε_v. The model parameters are the intrinsic permeability of the porous rock, k, the fluid viscosity and density, η_f and ρ_f, the specific storage of the saturated rock, $S_\epsilon = \phi\chi_f + (\alpha_B - \phi)\chi_s$, where χ_f and χ_s are, respectively, the fluid and solid compressibilities, and the rock porosity, ϕ. The Biot coefficient is α_B. Neglecting fluid accelerations and relative fluid-solid accelerations, the internal equilibrium equation for the solid is given by:

$$\rho_b \ddot{\mathbf{u}} = \nabla \cdot \sigma + \rho_b \mathbf{g}, \tag{2}$$

where ρ_b is the bulk density of the saturated rock. The Cauchy stress tensor is decomposed as the sum of an inviscid (elastic) part plus a viscoelastic one:

$$\sigma' = \sigma'_{invis} + \sigma'_{vis}. \tag{3}$$

We adopt a Saint Venant–Kirchhoff hyperelastic material for the inviscid part whose stress stensor is written in terms of the infinitesimal strain tensor as:

$$\sigma'_{invis} = \lambda \mathrm{tr}\,(\varepsilon)\,\mathbf{I} + 2G\varepsilon, \tag{4}$$

where G is the shear modulus and λ is the first Lamé constant, $\lambda = E\nu/(1+\nu)(1-2\nu)$, where E is the Young modulus and ν the Poisson ratio. For a Kelvin–Voigt material, the viscous component of the Cauchy stress tensor reads:

$$\sigma'_{vis} = \frac{\eta_{kv}}{G}\dot{\sigma}'_{iso}. \tag{5}$$

The total stress tensor is given by:

$$\sigma = \sigma'_{invis} + \sigma'_{vis} - \alpha_B p\mathbf{I} = \sigma' - \alpha_B p\mathbf{I}. \tag{6}$$

Finally, neglecting cohesion, the fault frictional strength is given by:

$$\tau_f = \begin{cases} -\mu\sigma'_n, & \sigma'_n < 0 \\ 0, & \sigma'_n \geq 0, \end{cases} \tag{7}$$

where σ'_n is the effective normal compression on the fault, and μ is the friction coefficient. The effective contact pressure σ'_n is given by $\sigma'_n = p - T_n$, with T_n being the contact pressure between the fault edges (compressive pressures are positive), and p is the fault pore pressure. Its value is chosen as the maximum on both sides of the fault, $p = \max(p^-, p^+)$ (Jha & Juanes, 2014). The fault remains locked when the shear stress acting on the fault is lower than the frictional strength, $< \tau_f$; otherwise, it slips. We model flow across the fault through a simple mass flux exchange between the two contact surfaces defining the fault. Denoting by $p^\pm$ the pressures on either side of the fault and by $\mathrm{q}_i n^\pm$ the inward mass fluxes per unit area, we approximate the latter through an effective fault transmissibility, T_f, as $q_i n^- = T_f(p^+ - p^-)$, and $q_i n^+ = T_f(p^- - p^+)$. The above jump condition couples the pore pressures on both sides of the fault, allowing a transition from essentially no-flow ($T_f \to 0$) towards pressure continuity ($T_f \to \infty$) as T_f increases. In this study, we consider small transmissibility, $T_f = 10^{-14}$ s/m, so that pressure diffusion across the fault is negligible for the time scales studied.

The fault constitutive behaviour is governed by the Dieterich–Ruina aging law, where the friction coefficient evolves according to:

$$\mu = \mu_0 + a\ln\left(\frac{V}{V_0}\right) + b\ln\left(\frac{V_0\theta}{D_c}\right), \tag{8}$$

and the state variable is as follows:

$$\frac{d\theta}{dt} = 1 - \frac{V\theta}{D_c}. \tag{9}$$

In the above equations, V is the slip speed, and (μ_0, a, b, D_c, V_0) are model parameters. For slip-weakening faults the coefficient of friction evolves from its static value, μ_s, to the

dynamic one, μ_d, over a characteristic distance, d_0, as

$$\mu = \begin{cases} \mu_s - (\mu_s - \mu_d)|d|/d_0, & |d| < d_0 \\ \mu_d, & |d| > d_0, \end{cases} \tag{10}$$

where $|d|$ is the total accumulated slip.

S2. Simulation setup

We simulate earthquakes in poroelastic media using two configurations and triggering mechanisms:

1. For Figures 1–4 of the main text: Earthquakes triggered by fluid injection near a strike-slip fault. The injection-induced model is a 2-D horizontal domain with a plane-strain poroelastic medium and an impermeable fault with rate-and-state friction, as the one described in [1] (Supplementary Figure S1). The fault is locked at the beginning of injection, with acting shear stress and frictional strength determined by anisotropic far-field tectonic stresses, $\sigma_x > \sigma_y$. Fluid injection near the center of the fault increases the pore pressure, triggering an earthquake when the acting shear stress equals frictional strength. We control the rupture propagation speed by varying the released stress during rupture (changing the confining stresses (σ_x, σ_y) and their ratio, as well as rock stiffness and poroelastic coupling (through the total system compressibility or specific storage, S_ϵ, and the Biot coefficient, α_B). The main field variables we use to characterize dynamic ruptures are the pore pressures, displacements, and accelerations. We also track the evolution of pore pressures on both sides of the fault, slip speed, and accumulated slip. The latter is used to estimate rupture propagation speed (Section S3). The model parameters used in the calculations leading to Figures 2, 3, 4a, and 4b of the main text are summarized in Tables S1-S3, respectively.

2. For Figures 5 of the main text: Earthquakes triggered by tectonic loading. We use this idealized scenario to investigate the validity of the Andrews' seismic ratio when computed using effective normal stresses. The tectonic model is a 2D horizontal domain that encloses a 4 km length strike-slip fault parallel to the x-axis, with a plane-strain poroelastic medium and an impermeable fault with a slip-weakening friction (Supplementary Figure S2). We generate the initial conditions of confinement (normal stress acting on the fault by imposing a displacement in the y-direction along the upper and lower boundaries, negative and positive respectively. This imposed displacement leads to an initial effective normal stress ($\sigma'_{n,0} \approx 9.15$ MPa). From the initial state of normal confinement, we gradually increase the applied shear stress keeping by imposing a time dependent x-displacement along the horizontal boundaries (a shear rate), and a triangular law on the vertical boundaries (Supplementary Figure S2). The gradual increase of shear stress acting on the fault eventually leads to the nucleation of earthquakes at a small weaker patch at the center of the fault. We trigger failure by reducting assuming a smaller static friction coefficient at a small seed region around the center of the fault. The model parameters used in the calculations leading to Figure 5 of the main text are summarized in Table S4.

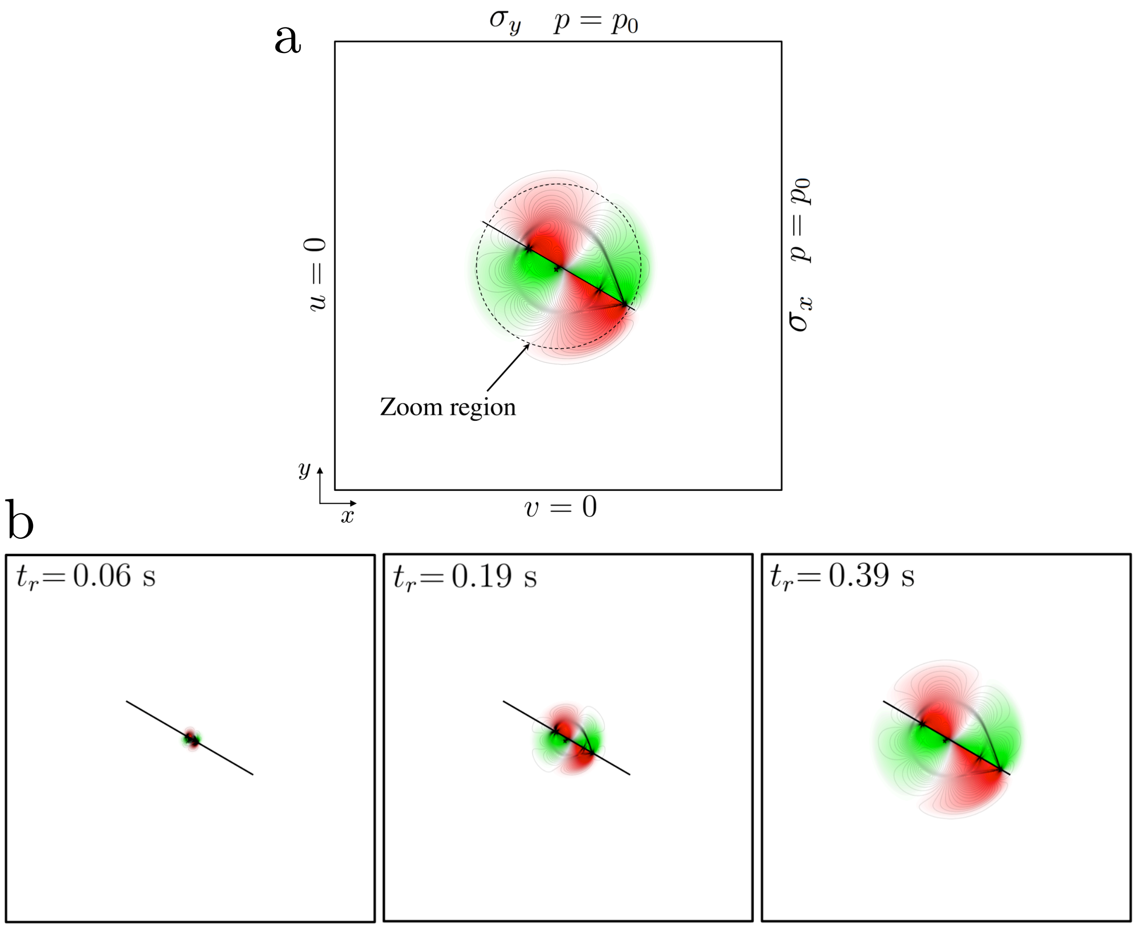

Figure S1: Schematic and boundary conditions for earthquakes triggered by fluid injection.

Boundary conditions

	u (m)	v (m)
1	10^{-7} t	-2
2	-10^{-7} t	2
3	10^{-7} t (Y / 2500 - 1)	−2Y / 2500 + 2
4	10^{-7} t (Y / 2500 - 1)	−2Y / 2500 + 2

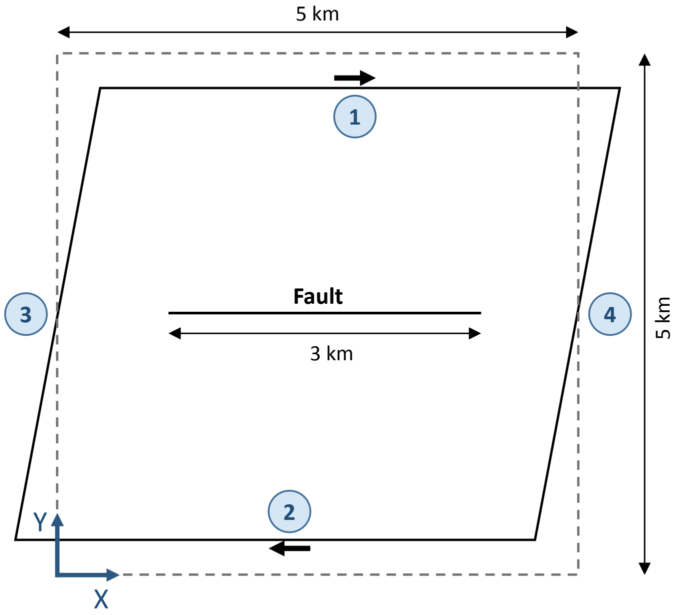

Figure S2: Schematic and boundary conditions of the tectonic model, with u and v being the imposed boundary displacement along x and y directions, respectively.

S3. Estimating the speed of rupture propagation

We compute the rupture propagation speed during a simulation, $V_R(t)$, using simple first-order differencing of the front positions over time (Supplementary Figure S3 below). The position of the rupture front is calculated from the profile of accumulated slip along the fault. It is defined as the coordinate along the fault of the first node whose accumulated slip is larger than a threshold value (we take 10^{-4} m). We then compare rupture front positions between time steps separated by a lag that reduces differencing noise (i.e. we compute the rupture speed at step i by comparing the front position at step i and the position at step $i-50$), and divide the distance travelled by the elapsed time:

$$V_{R,i} = \frac{X_{f,i} - X_{f,i-50}}{t_i - t_{i-50}}, \tag{11}$$

where $V_{R,i}$ is the rupture propagation speed at time level t_i, and $X_{f,i}$ is the position of the rupture front at that same time level (coordinate along the fault). Rupture speeds calculated using finite differences are necessarily oscillatory. To reduce high-frequency oscillations in the calculation, we filter the raw rupture speeds using a moving average (Supplementary Figure S3 below). In Figure S3 we show two examples of evolution of front speed (these correspond to two data points in Figure 4 of the main text, one sub-Rayleigh and one supershear). We show the variation of the relative speeds, $V_{R,i}/C_{S,0}$, throughout the simulation, for $E = 50\,\text{GPa}$ and $\alpha_B = 0.2$ (panels **a** and **b**), and $E = 50\,\text{GPa}$ and $\alpha_B = 0.9$ (panels **c** and **d**), which propagate, respectively, in sub-Rayleigh and in supershear regimes. In panels (**b**) and (**d**) we highlight the average value of rupture speed that we has been considered for Figure 4, $\bar{V}_R/C_{S,0}$. Figure S4 shows the evolution in time of rupture speeds for all the simulation cases reported in Figure 4, where we set four values of the Young modulus, $E = 35, 50, 75,$ and $90\,\text{GPa}$ and several values of the Biot coefficient α_B, which characterize the parametric transition from sub-Rayleigh to supershear propagation. For reference, we show here again Figure 4a of the main text, which summarizes the rupture speeds as a function of rock properties E and α_B (shown here as Figure S5).

S4. Impact of grid resolution on rupture speed

To assess the impact of grid resolution on the reported behavior of the rupture speeds, we performed a refinement study for a set of simulations from those shown in Figure 4 of the main manuscript, namely those with $E = 75\,\text{GPa}$ and several values of the Biot coefficient (Figures S6 and S7). We used computational grids with grid sizes along the fault of $\delta s = 2, 4,$ and $10\,\text{m}$. Figure S6 compares the evolution of rupture speed for four values of the Biot coefficient and the three refinement levels. While the different grid resolutions yield slightly different evolution of the rupture speed, the asymptotic rupture speeds, which are the relevant output for our study, are quite robust. The main differences among grid refinement levels arise at the parametric transition to supershear rupture ($\alpha_B = 0.55$, Figure S7). Here the coarse and fine level lead to subshear rupture while the intermediate grid level yields a supershear rupture. We believe this behavior is consistent with the idea that the parametric transition is truly a bifurcation with discontinuous behavior, rather than a smooth transition to supershear as the parameter (the Biot coefficient) increases.

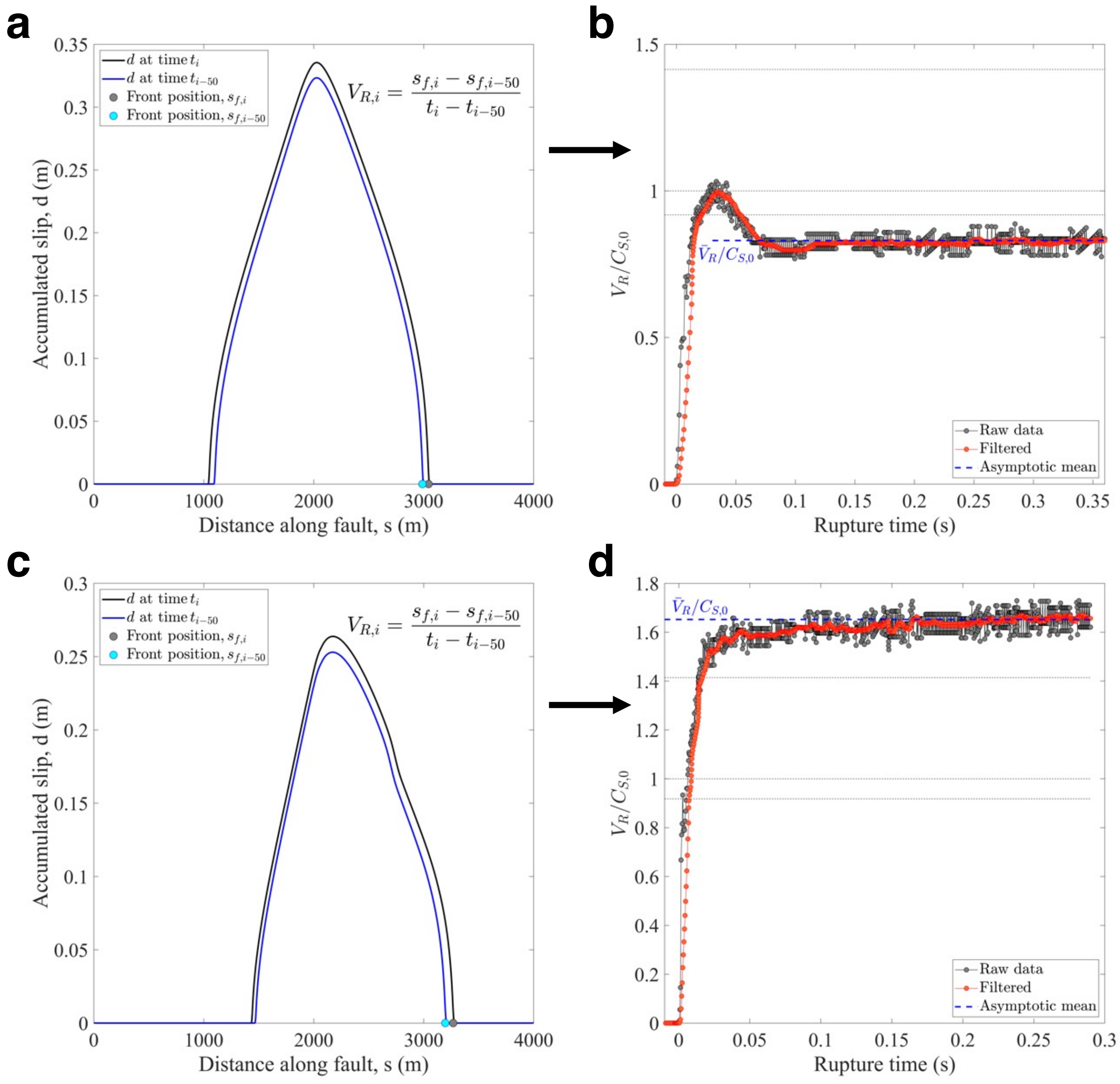

Figure S3: Calculation of rupture propagation speeds. We estimate rupture speeds using first-order time differencing of the front locations along the fault, as determined from the evolution of the profile of accumulated slip (panels **a** and **c**). The rupture speeds calculated using finite differences are noisy, so we estimate average speeds using moving averages (panels **b** and **d**). In this figure we show the calculation of rupture speeds for two of the data points shown in Figure 4 of the main text: those with parameters $E = 50\,\mathrm{GPa}$ and $\alpha_B = 0.2$ (panels **a** and **b**), and $E = 50\,\mathrm{GPa}$ and $\alpha_B = 0.9$ (panels **c** and **d**).

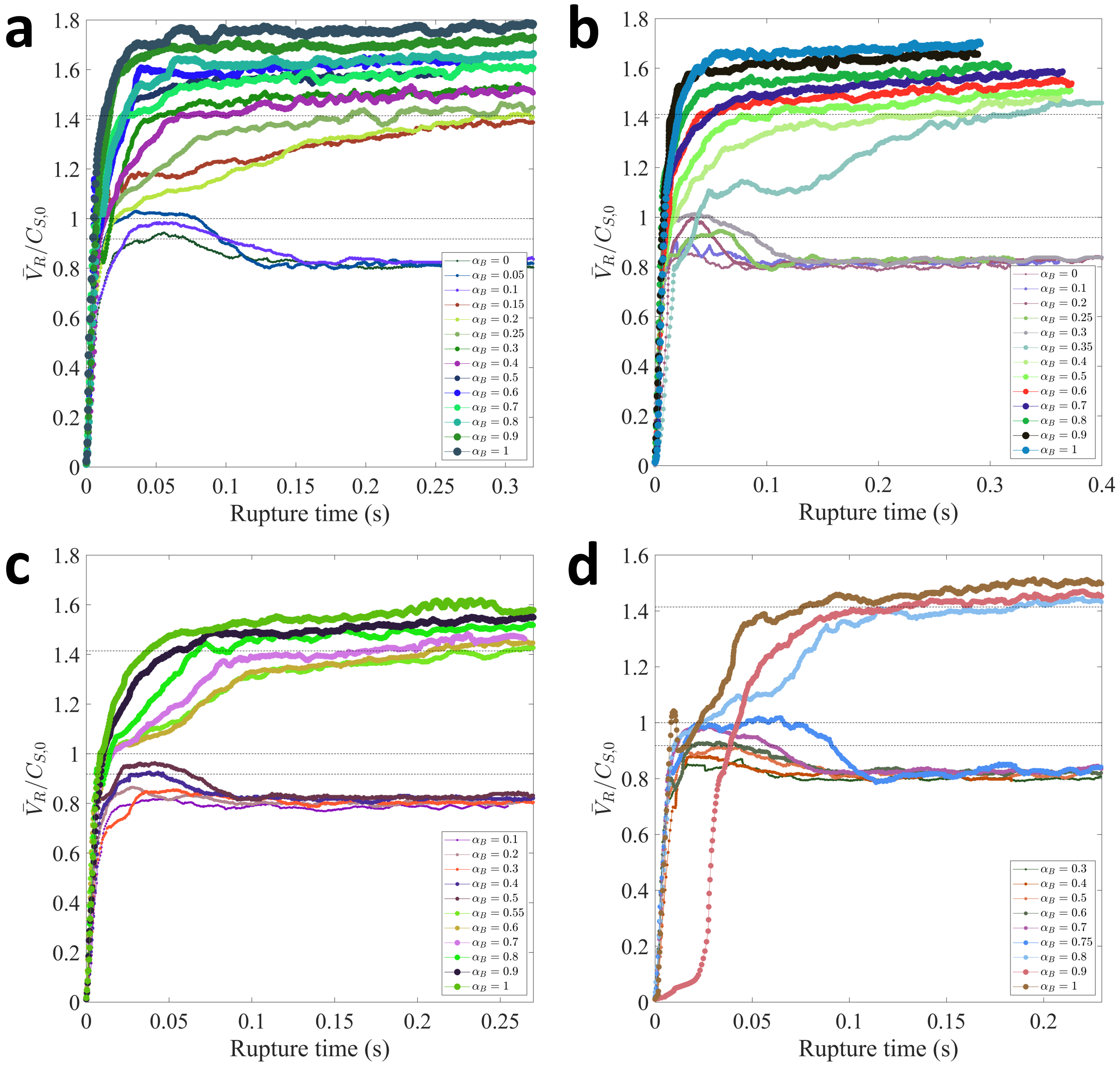

Figure S4: Sample evolutions of rupture front speed. Here we show the evolution of rupture speed during rupture, for the simulation cases shown in Figure 4 of the main text. Each panel shows the evolution of rupture speed for a certain value of the Young modulus, E, and several values of the Biot coefficient, α_B: $E = 35\,\text{GPa}$ (panel **a**), $E = 50\,\text{GPa}$ (panel **b**), $E = 75\,\text{GPa}$ (panel **c**), and $E = 90\,\text{GPa}$ (panel **d**).

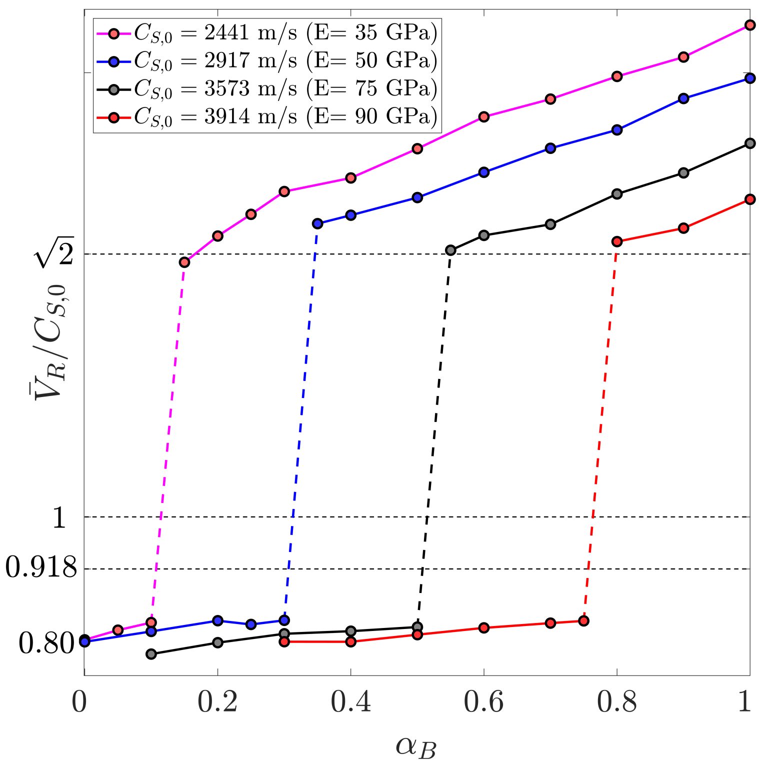

Figure S5: Summary of rupture front speeds from the previous figure (Fig. S4). This is also shown as Figure 4a of the main text.

S5. Estimating the nominal and effective seismic ratios

To illustrate the calculation of the seismic ratio, we study the changes in frictional strength and on applied shear stress during rupture propagation in earthquakes driven by tectonic loading (Supplementary Figure S4), For our slip-weakening fault, the Andrews seismic ratio, S, is simply defined as [2, 3]:

$$\mathrm{S} = \frac{\mu_s\ \sigma_n' - \tau_a}{\tau_a - \mu_d\ \sigma_n'} = \frac{\mathrm{S1}}{\mathrm{S2}}, \tag{12}$$

where μ_s and μ_d are the static and dynamic friction coefficients, σ_n' is the effective normal stress, and τ_a is the applied shear rate. In order to envisage the seismic ratio, we have renamed the numerator and the denominator as S1 and S2, respectively. In Supplementary Figure S4 we compare the profiles of shear stress and frictional strength along the fault, for the same overall simulation parameters, and two contrasting cases in terms of poroelastic coupling: the purely elastic case α_B=0 (panel a) and the poroelastic case with α_B=0.95 (panel b). Calculating the seismic ratio for the elastic case is immediate (Supplementary Figure 4a), and the sub-Rayleigh to supershear transition (Supplementary Figure 6) is associated to a seismic ratio of S=1.77 (as noticed by Andrews [2]). The effective seismic ratio, S′, is defined using effective quantities at the front in Supplementary Figure 4b, as: $\mathrm{S}' = \frac{\mathrm{S1}}{\mathrm{S2}'}$.

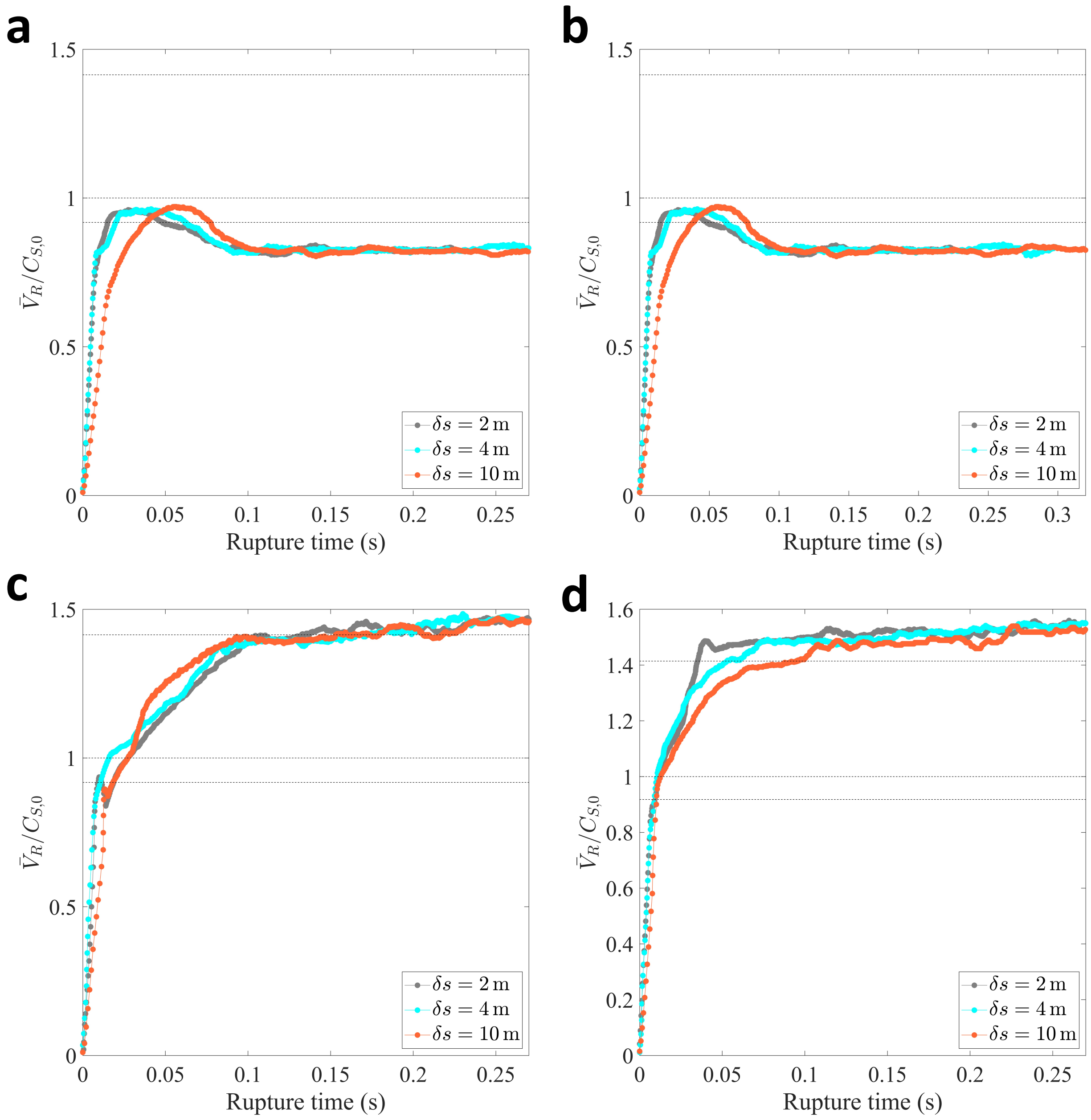

Figure S6: Evolution of rupture speed for four values of the Biot coefficient and the three refinement levels. The Young modulus is $E = 75\,\text{GPa}$, and the Biot coefficient is $\alpha_B = 0.2$ (panel **a**), $\alpha_B = 0.5$ (panel **b**), $\alpha_B = 0.7$ (panel **c**), and $\alpha_B = 0.9$ (panel **d**).

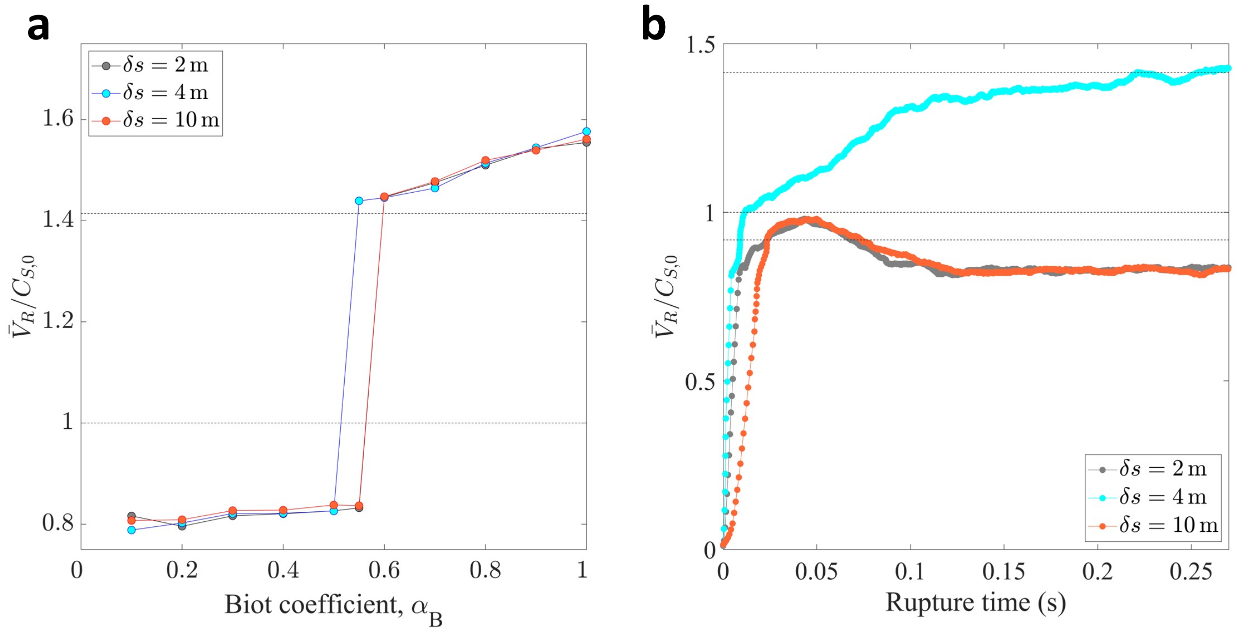

Figure S7: Summary of rupture speeds for $E = 75\,\mathrm{GPa}$ and the three refinement levels (panel **a**), and evolution of rupture speeds for $\alpha_B = 0.55$ (panel **b**).

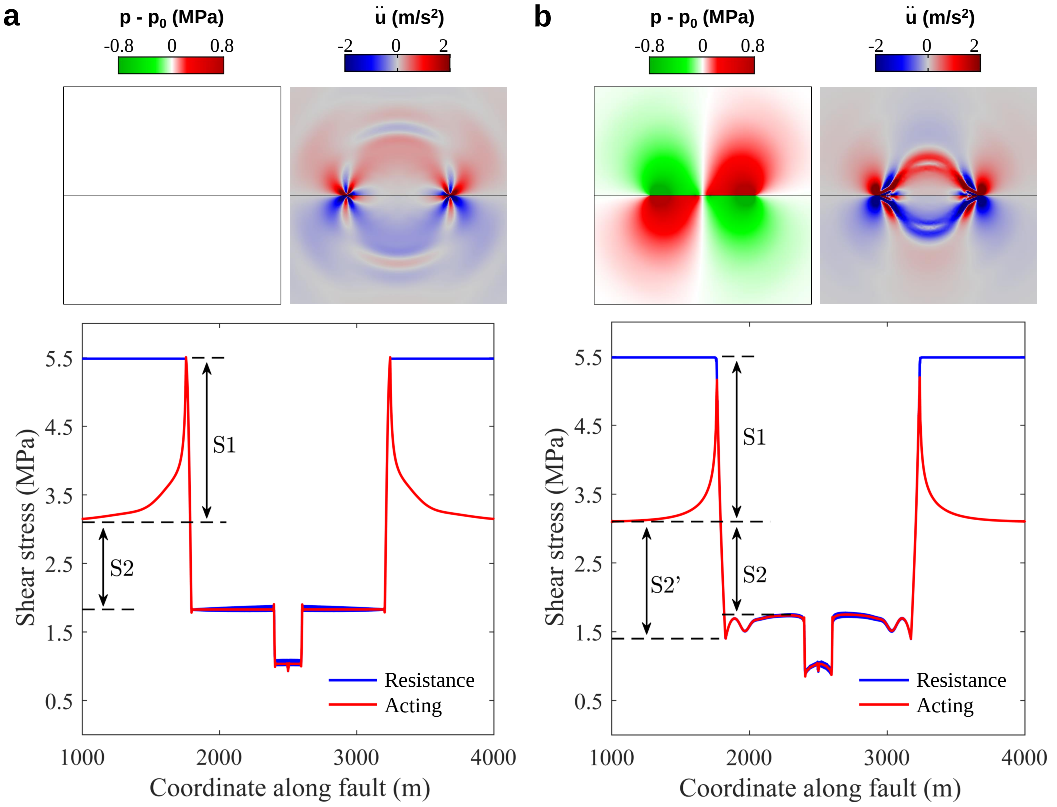

Figure S8: Acting shear stress and shear resistance along the fault during the rupture of a model with $\mu_d = 0.2$ for an elastic (panel a) and for a poroelastic case (panel b). Above, snapshots of overpressures and accelerations at the instant of rupture. S1, S2 and S2' are the quantities involved in the calculation of the seismic ratio.

Table S1: Parameters for Figure 2 (earthquakes triggered by fluid injection)

**Parameter**	**Value**	**Unit**	**Description**
L	4	km	Fault length
σ_x	20	MPa	x-confinement (effective stress)
σ_y	10	MPa	y-confinement (effective stress)
A	30	deg	Fault orientation with respect to the x-axis
q	0.17	kg/(s·m)	Injection flow rate
E	20	GPa	Young's modulus of the rock
ν	0.25	–	Poisson ratio of the rock
η_{kv}	10	MPa·s	Kelvin-Voigt solid viscosity
ρ_s	2500	kg/m^3	Dry density of the rock
ρ_b	2350	kg/m^3	Bulk density of the saturated rock
ρ_f	1000	kg/m^3	Density of the fluid
η_f	0.001	Pa·s	Dynamic viscosity of the fluid
χ_f	$4{\cdot}10^{-10}$	Pa^{-1}	Compressibility of the fluid
k	10^{-14}	m^2	Intrinsic permeability
ϕ	0.1	–	Porosity
α_B	0 - 0.95	–	Biot's coefficient
μ_0	0.6	–	Reference friction coefficient
a	0.005	–	Direct effect parameter
b	0.02	–	Friction evolution parameter
D_c	0.0002	m	Characteristic slip distance
V^*	10^{-9}	m/s	Reference slip velocity

Table S2: Parameters for Figure 3 (earthquakes triggered by fluid injection)

**Parameter**	**Value**	**Unit**	**Description**
L	4	km	Fault length
σ_x	20	MPa	x-confinement (effective stress)
σ_y	10	MPa	y-confinement (effective stress)
A	30	deg	Fault orientation with respect to the x-axis
q	0.17	kg/(s·m)	Injection flow rate
E	20	GPa	Young's modulus of the rock
ν	0.25	–	Poisson ratio of the rock
η_{kv}	10	MPa·s	Kelvin-Voigt solid viscosity
ρ_s	2500	kg/m^3	Dry density of the rock
ρ_b	2350	kg/m^3	Bulk density of the saturated rock
ρ_f	1000	kg/m^3	Density of the fluid
η_f	0.001	Pa·s	Dynamic viscosity of the fluid
χ_f	$2{\cdot}10^{-10}$ - $2{\cdot}10^{-8}$	Pa^{-1}	Compressibility of the fluid
k	10^{-14}	m^2	Intrinsic permeability
ϕ	0.1	–	Porosity
α_B	0.95	–	Biot's coefficient
μ_0	0.6	–	Reference friction coefficient
a	0.005	–	Direct effect parameter
b	0.02	–	Friction evolution parameter
D_c	0.0002	m	Characteristic slip distance
V^*	10^{-9}	m/s	Reference slip velocity

Table S3: Parameters for Figure 4 (earthquakes triggered by fluid injection)

**Parameter**	**Value**	**Unit**	**Description**
L	4	km	Fault length
σ_x	130	MPa	x-confinement (effective stress)
σ_y	50	MPa	y-confinement (effective stress)
A	30	deg	Fault orientation with respect to the x-axis
q	0.255	kg/(s·m)	Injection flow rate
E	35, 50, 75, 90	GPa	Young's modulus of the rock
ν	0.25	–	Poisson ratio of the rock
η_{kv}	1	MPa·s	Kelvin-Voigt solid viscosity
ρ_s	2500	kg/m^3	Dry density of the rock
ρ_b	2350	kg/m^3	Bulk density of the saturated rock
ρ_f	1000	kg/m^3	Density of the fluid
μ_f	0.001	Pa·s	Dynamic viscosity of the fluid
χ_f	$4{\cdot}10^{-10}$	Pa^{-1}	Compressibility of the fluid
k	10^{-14}	m^2	Intrinsic permeability
ϕ	0.1	–	Porosity
α_B	[0,1]	–	Biot coefficient
μ_0	0.6	–	Reference friction coefficient
a	0.005	–	Direct effect parameter
b	0.012	–	Friction evolution parameter
D_c	0.0002	m	Characteristic slip distance
V^*	10^{-9}	m/s	Reference slip velocity

Table S4: Parameters for Figure 5 (earthquakes triggered by tectonic loading)

**Parameter**	**Value**	**Unit**	**Description**
L	3	km	Fault length
σ_x	3.2	MPa	x-confinement (effective stress)
σ_y	9.6	MPa	y-confinement (effective stress)
τ_a	0.1596	Pa/s	Applied shear rate
E	10	GPa	Young's modulus of the rock
ν	0.25	–	Poisson ratio of the rock
η_{kv}	1	MPa·s	Kelvin-Voigt solid viscosity
ρ_s	2500	kg/m^3	Dry density of the rock
ρ_b	2350	kg/m^3	Bulk density of the saturated rock
ρ_f	1000	kg/m^3	Density of the fluid
η_f	0.001	Pa·s	Dynamic viscosity of the fluid
χ_f	$4{\cdot}10^{-10}$	Pa^{-1}	Compressibility of the fluid
k	10^{-14}	m^2	Intrinsic permeability
ϕ	0.1	–	Porosity
α_B	0.95	–	Biot's coefficient
μ_s	0.6	–	Static friction coefficient
μ_d	0.1 - 0.27	–	Dynamic friction coefficient
d_0	0.05	m	Critical slip-weakening distance
V^*	10^{-9}	m/s	Reference slip velocity

References

[1] Pedro Pampillón, David Santillán, Juan Carlos Mosquera, and Luis Cueto-Felgueroso. Dynamic and quasi-dynamic modeling of injection-induced earthquakes in poroelastic media. *Journal of Geophysical Research: Solid Earth*, 123(7):5730–5759, 2018.

[2] D. J. Andrews. Rupture velocity of plane strain shear cracks. *Journal of Geophysical Research (1896-1977)*, 81(32):5679–5687, 1976.

[3] M. Mello, H.S. Bhat, A.J. Rosakis, and H. Kanamori. Identifying the unique ground motion signatures of supershear earthquakes: Theory and experiments. *Tectonophysics*, 493(3):297 – 326, 2010.
